# Interindividual and sex differences in resilience and vulnerability to post-traumatic stress disorder (PTSD): insights from animal models

**DOI:** 10.1186/s13293-025-00732-5

**Published:** 2025-07-01

**Authors:** Giulia Federica Mancini, Sebastiano Alfio Torrisi, Eva Myriam Goussivi Viho, Onno Cornelis Meijer, Gian Marco Leggio, Patrizia Campolongo

**Affiliations:** 1 Department of Physiology and Pharmacology, Sapienza University of Rome, P.le A. Moro 5, Rome, 00185 Italy; 2 Department of Biomedical and Biotechnological Sciences, University of Catania, Catania, 95123 Italy; 3 Department of Genes and Environment, Max Planck Institute of Psychiatry, 80804 Munich, Germany; 4 Department of Medicine, Division of Endocrinology, Leiden University Medical Center, Leiden, 2333 ZA The Netherlands; 5 Neuropharmacology Unit, IRCCS Fondazione Santa Lucia, Rome, 00143 Italy

**Keywords:** Experimental paradigms, Susceptibility, Rodents, Psychopathologies, Sex disparities

## Abstract

Stress triggers many responses including behavioral strategies to cope with the environment and to maintain homeostasis. Notably, the experience of stressful events is highly subjective. In fact, in susceptible individuals, primary adaptation responses can fail leading to maladaptive mechanisms and to the subsequent development of stress-related disorders (e.g., post-traumatic stress disorder; PTSD). However, the mechanisms underlying interindividual variability in stress adaptation are still to be elucidated. Animal models are widely recognized as essential scientific tools to understand the neurobiological underpinnings of stress susceptibility/resilience, and as tools to identify novel and personalized interventions to treat (and prevent) such disorders in humans. Experimental models have however several limitations, as validity criteria can be very problematic when modeling psychiatric disorders. Also, while sex dimorphism crucially contributes to the risk for stress-related diseases, several frequently used models overlooked sex differences in the interindividual variability in response to stress. In this review, we describe the interindividual and sex differences in susceptibility and resilience in stress-related disorders, with a particular focus on PTSD. Further, we examine aspects of animal models of PTSD that can be improved to obtain higher translational value.

## Abstract (plain language summary)

People respond to stress in different ways. While some recover quickly, others struggle and may develop conditions like post-traumatic stress disorder (PTSD). Understanding why some individuals are more vulnerable than others remain a major challenge. Animal models help researchers study the mechanisms behind stress resilience and susceptibility, as well as explore potential treatments. However, preclinical models fail to fully capture the complexity of psychiatric disorders like PTSD. In particular, sex differences are often overlooked, even though women are at higher risk for this pathology. This review discusses how both individual and sex differences influence PTSD risk and highlights ways to improve animal models to make them more relevant for developing effective, personalized therapies.

## Highlights

Distinguishing between susceptible and resilient subjects is crucial for identifying the mechanisms that drive PTSD development in vulnerable populations.

Animal models are essential for understanding PTSD, but their translational value is limited by the frequent lack of consideration for sex differences and individual variability in stress adaptation.

Sex differences significantly impact PTSD risk, yet female rodents have been historically overlooked in preclinical studies, highlighting the urgent need for more inclusive experimental designs.

Improving PTSD models to better reflect human variability, including both sex and interindividual differences, could facilitate the identification of reliable biomarkers and the development of personalized therapeutic strategies.

## Background

Stress was originally defined by Hans Selye, the founder of stress theory, as a nonspecific reaction of the body to any kind of stimulus [1]. According to this theory, stress is not limited to negative or unpleasant experiences, but encompasses any situation that requires adaptation or adjustment from an individual. A few decades later the concepts of “stressor” and “stress response” have been introduced. A stressor has been described as a physical or psychological event altering the dynamic equilibrium of an organism (homeostasis), and the stress response as the mechanisms, involving the neuroendocrine, autonomic, immune and metabolic systems, adopted to cope with stress and thus restore the physiological equilibrium [2, 3]. Stressful events are very frequent in humans throughout life, and they can have a profound impact on individuals [4, 5]. Of note, the way persons respond to these experiences can vary significantly. In fact, some persons (susceptible) develop stress-related disorders (e.g., post-traumatic stress disorder, PTSD) after stress/trauma exposure, while others (resilient) do not and - somehow - better deal with severe life challenges.

The biological sex of the individual is important to consider in the context of the interindividual variability in response to stress. Interestingly, it has been demonstrated that women are more likely to develop stress-related disorders compared to men [6, 7]. This could be due to several factors, including the fact that males and females are exposed to different types of stressors during their lifetime, the distinct perception of the stress, the different structure and function of the stress response system’s components as well as limbic regions involved in the stress response, and the interplay between stress and sex hormones. Even though it is clear that interindividual and sex differences exist in stress/trauma response [8–15], the neurobiological and causative mechanisms underlying such variabilities still remain to be clarified; this represents a significant challenge in the medical field as it hinders the ability to develop suitable treatment and prevention strategies for stress-related disorders.

The use of experimental models of psychiatric disorders could be beneficial in this context. The translational benefit of animal models has been already argued for based relevant neuroanatomical similarities between rodents and humans [16, 17]. However, modeling psychiatric diseases, and in particular PTSD, at the preclinical level can be very difficult. PTSD is a chronic psychiatric disorder with no well-defined etiopathogenesis and thus neurobiological underpinnings, and no gold standard therapy currently exists, which challenges the reproduction of all the three criteria (‘face validity’, ‘construct validity’ and ‘predictive validity’) that need to be considered when studying a psychiatric disorder in animal models.

Here, we provide a comprehensive overview of the variability observed among individuals in their response to stress/trauma and the subsequent development of mental diseases, with a particular focus on PTSD. Furthermore, we delve into the impact of sex differences (e.g., hormonal, neurobiological factors) contributing to the different vulnerability (or resilience) for the development of stress-related disorders. Then, we examine existing animal models of PTSD by pointing out their limitations in modeling the complexity of the human pathology in order to improve these aspects and thus to obtain higher translational impact.

## Physiological stress response system

The hypothalamic-pituitary-adrenal (HPA) axis, working in concert with systems such as the autonomic nervous system and the hypothalamic-pituitary-gonadal (HPG) axis, plays an important role in regulating the stress response. Upon detection of stressors, the hypothalamic paraventricular nucleus (PVN) neurons produce the corticotropin-releasing hormone (CRH), which stimulates the anterior pituitary gland to produce and release the adrenocorticotropic hormone (ACTH) which in turn induces the secretion of glucocorticoids (primarily cortisol in humans and corticosterone in rodents) from the adrenal cortex. Once circulating in the bloodstream, glucocorticoids can regulate many processes in the periphery (e.g., carbohydrate, lipid and protein metabolism, and inflammation) and - based on their ability to cross the blood brain barrier - in the brain (e.g., learning and memory, behavioral responsiveness and mood) [18–20]. Glucocorticoids can mediate these effects via binding to glucocorticoid and mineralocorticoid receptors (GR and MR, respectively) which predominantly behave as intracellular receptors and belong to the ligand-dependent transcription factor family [21, 22]. MRs have a 10-fold higher affinity for glucocorticoids compared to GRs, thus suggesting that these receptors exert different roles in the regulation of the HPA axis [23, 24]. In fact, MRs are thought to be substantially occupied at basal hormone levels, while GRs are activated at the circadian peak of glucocorticoids release, and following stress exposure [25]. This has led to the notion that MRs play an important role in the regulation of the HPA axis basal activity and its activation, and GRs modulate stress recovery and adaptation [4, 26, 27]. Of note, via binding through their receptors located in the pituitary gland and across several brain regions, glucocorticoids also regulate their own release via a negative feedback mechanism. This establishes appropriate glucocorticoid release under basal conditions as well as the termination of the HPA axis activation during stress recovery. Following stressful/traumatic events, potential dysfunctions of the negative feedback and dysregulations of the actions of GRs/MRs can induce maladaptive mechanisms at the cellular and network level which in turn can increase the risk for developing stress-related disorders [4, 21].

## Adaptive versus maladaptive mechanisms: stress-related disorders

Under stressful conditions, many systems are involved in processes necessary to restore the homeostasis (adaptation). However, these adaptive mechanisms cannot be sustained for a long time. In fact, when stress is excessive or prolonged, the restoration of homeostasis is challenged. This condition comes with allostatic load, which refers specifically to the long-term strains as the consequence of an excessive or prolonged activation of the adaptive mechanisms. In some individuals the adaptive mechanisms can become dysfunctional (maladaptive mechanisms), and this can induce an enhanced susceptibility to the development of stress-related disorders [4, 5]. Of note, not all individuals exposed to a severe acute stress or a prolonged stress will eventually develop psychopathologies. For example, after a traumatic or life-threatening experience only a subset of subjects will develop PTSD (susceptible), whilst most of the trauma victims will fully recover after the first acute physiological response (resilient). The important question of why some individuals are more susceptible to developing stress-related disorders after experiencing a stressful/traumatic event still remains an open question.

The existence of interindividual differences in the stress response depends on a combination of several interacting factors, including genetics, physiological and psychological history of the individual, subjective interpretation of the stressful situation, life stage during which the stress occurs, environmental influences and biological sex [28, 29]. Regarding the genetic predisposition, it has been established that polymorphisms in several genes (e.g., serotonin transporter *SLC6A4* or *NR3C2*) are involved in the susceptibility for stress-related disorders [30–32]. Previous life experiences play an important role in the response to stress. Regarding physiological systems, interindividual differences in stress responses can vary based on factors such as the autonomic nervous system and the HPA axis [33]. The function or activity of these systems can shape and/or reflect an individual’s coping strategies in response to stress [34], thereby influencing their susceptibility to developing stress-related disorders. Moreover, the perception of a stressful experience is a subjective component of the stress response, as it is based on the unique life history of an individual. Growing evidence demonstrates that early-life exposure to stressful situations can enhance the risk for the development of stress-related disorders in adulthood and alters the ability to cope with additional challenges experienced later in life. For example, trauma during early-life is one of the major risk factors for PTSD development [35]. However, the effects of stress also vary depending on its type and length, and environmental factors can modulate these outcomes. Interestingly, a previous published work demonstrated that variations in maternal care significantly affect stress responsivity in the adult offspring [36]. This is in line with the concept that the exposure to two different stressors in two different life periods may induce either susceptibility or resilience towards stress-related disorders [37–40], and that these outcomes could differ according to the timing and the context of the first stressor (or ‘hit’) in rodents [29]. Another aspect regarding the interindividual differences in the stress susceptibility/resilience that needs to be considered is the biological sex. This is illustrated by the effects that sex hormones can have on the HPA axis, but extends to other aspects of the stress response [41]. Below we expand on the role of biological sex and sex hormones in stress-related diseases.

## Stress- and sex- hormones crosstalk

It has been established that sex differences in the development of stress-related disorders (e.g., PTSD) occur, and that this can be related to the socio-cultural disparities between men and women as well as to the differences in their biological mechanisms at both cellular and molecular levels [6, 42]. Sex hormones are most often considered as the mediators of biological causes of these sex differences. Sex hormones include estrogens (e.g., estradiol), progesterone, and androgens (e.g., testosterone and its metabolite dihydrotestosterone) which bind to estrogen receptors (estrogen receptor alpha and beta; ERα and ERβ), progesterone receptors (PRs), and androgen receptors (ARs), respectively. These nuclear receptors belong to the same receptor subfamily as GRs and MRs, and they are widely expressed in numerous brain areas [43].

Sex differences in the endocrine stress response are firstly related to the differences existing at each level of the HPA axis (PVN, pituitary and adrenal glands), as well as the limbic brain regions involved in its regulation. Secondly, the stress response and the activity of the HPA axis can be altered in adulthood via functional crosstalk with the hypothalamic-pituitary-gonadal (HPG) axis [44].

Intrinsic differences in the HPA-axis include that during adolescence, which is a highly critical developmental and stress-sensitive window, the volume of the adrenal glands under normal conditions is bigger in females compared to male rats [45]. Male and female rats also differ in CRH receptors type 1 (CRHR1) and type 2 (CRHR2) modulation with sex hormones implicated in these effects. CRH binding to CRHR1 is higher within the amygdala and cortex in female rats, whereas in males CRH predominantly binds to CRHR2 within the amygdala and hypothalamus [46]. Moreover, GR expression within the pituitary, hippocampus and PVN is lower in female rats compared to males [44, 47, 48].

Regarding the functional crosstalk between the HPA and the HPG axes, fluctuations of estradiol levels during the estrous cycle in female rodents play an important role in the sex-biased activation of the HPA axis [49]. Generally, the estrous cycle in rodents lasts four days and it includes proestrus, estrus, metestrus and diestrus phases which are characterized by different estradiol and progesterone plasma concentration [50]. Previous studies demonstrated that, at both basal and stressful conditions, female rodents with relatively higher levels of estradiol and progesterone showed higher levels of ACTH and corticosterone with respect to females having low estradiol levels, and that the HPA axis activation in females with low estradiol levels is similar to that one of male subjects [44, 51, 52]. Of note, it is important to consider that such estradiol-induced HPA axis activation also depends on progesterone [53]. Viau and Meaney (1991) investigated the role of sex hormones in the stress response, by subjecting ovariectomized female rats to an acute stressor after treatment with physiological levels of estradiol with or without progesterone. They found that ovariectomized female rats treated with estradiol alone exhibited higher HPA axis activation upon stress exposure compared to female rats treated with both estradiol and progesterone, suggesting that progesterone could modulate the effects of estradiol on the HPA axis [52]. Altogether, these results suggest that estradiol increases HPA axis activity. However, there are some conflicting results showing that estradiol treatment can also inhibit the HPA axis [54, 55]. These estradiol-induced opposite effects may be explained by the differences between the two estrogen receptors ERα or ERβ which exert their function on the HPA axis through different mechanisms. In fact, it has been demonstrated that HPA axis activity can be increased by the selective modulation of ERα, and decreased by the selective modulation of ERβ [56].

While estrogens (generally) increase HPA axis activity, androgens dampen it [57]. In a previous study it has been shown that gonadectomized male rats treated with low dose of testosterone exhibit higher stress-induced HPA axis activation compared to gonadectomized male rats treated with a higher dose of testosterone [52]. This inhibitory effect induced by testosterone on the HPA axis is caused by the involvement of its metabolite dihydrotestosterone. In fact, central inhibition of the 5α-reductase enzyme, which converts testosterone to dihydrotestosterone, led to an increase in stress-induced secretion of glucocorticoids in male rats, which is counteracted by the re-introduction of dihydrotestosterone, but not testosterone [58]. Testosterone can also reduce ACTH and corticosterone release via direct inhibition of PVN neuronal activation in rats [59]. All these results indicate that androgens inhibit HPA axis activity and that the testosterone-induced effects are strongly dependent on the 5α-reductase function in the brain, and PVN neuronal activity. Differences in the endocrine (HPA) stress response may in large measure be explained by the regulation of corticosteroid binding globulin (CBG) in the liver. Estrogens stimulate CBG expression [52], while androgens, via the AR, suppress it. Increased circulating CBG leads to a lower fraction of ‘free’ or bioavailable cortisol and corticosterone. This smaller free fraction can lead to reduced negative feedback in the HPA axis, and this primes the system for an increased endocrine stress response [60]. Interestingly, the differential effects of gonadal hormones on HPA axis function are also reflected at the behavioral level. It has been shown that exposure to the same stressor facilitates associative learning in male rats, while impairing it in females, with these effects being mediated by sex-specific hormonal pathways [61, 62]. These findings suggest that hormonal modulation contributes not only to physiological but also to behavioral (e.g., fear memory) differences in stress responses between sexes.

Importantly, while much research has focused on the influence of gonadal hormones on HPA axis function and stress responses, emerging evidence suggests that genetic sex (i.e., XX or XY chromosomes) may also play a role in sex differences in stress susceptibility, although the extent of this contribution can vary depending on the biological context. For example, one study in primary human cell cultures found negligible intrinsic (chromosome-dependent) sex differences in the transcriptional response to cortisol [63]. Conversely, at the preclinical level, the Four Core Genotypes (FCG) model, which dissociates gonadal sex (testes vs. ovaries) from genetic sex (XX or XY), provides a powerful tool to disentangle the relative contributions of hormones, chromosomes, and their interactions, thereby elucidating mechanistic basis for biological sex differences [64]. For example, a recent study using the FCG model investigated the effects of gonadal and genetic sex on stress-induced phenotypes (e.g., anhedonia- and depressive- like behaviors). The results showed that both gonadal and genetic sex contribute to increased stress vulnerability, highlighting the importance of considering both factors in understanding sex differences in response to stress [65]. These findings suggest that mechanisms beyond hormonal regulation, such as X-linked genes that escape inactivation or Y-linked genes, may modulate HPA axis activity and influence susceptibility to stress-related disorders like PTSD. This has critical implications for clinical research, where sex differences are often studied within a binary male/female framework, potentially overlooking the biological variability present in intersex individuals (i.e., individuals born with sex characteristics that do not fit typical medical definitions of male or female) [66]. Although this area remains less explored, such insights could be essential for developing inclusive, personalized approaches in mental health care and may reveal novel biological mechanisms underlying stress-related disorders beyond traditional sex classifications. Therefore, a comprehensive understanding of sex differences in stress biology should integrate both gonadal (hormonal) and chromosomal (genetic) influences, as well as their complex interplay under diverse physiological and environmental conditions.

## PTSD and sex differences

Exposure to a traumatic and/or life-threatening event may induce inadequate or counteractive coping mechanisms leading to (an increased risk for) PTSD development in susceptible individuals [4, 5]. PTSD is listed as a ‘trauma and stressor-related disorder’ in the latest edition of the Diagnostic and Statistical Manual of Mental Disorders (DSM-5) [67]. PTSD is a chronic and complex psychiatric disorder; patients affected by this pathology are in fact characterized by a plethora of symptoms such as hyperarousal, hypervigilance, increased anxiety, reduced sociability and cognitive dysfunctions (e.g., over-consolidation, increased retrieval and impaired extinction of the traumatic memory) [67]. PTSD is highly debilitating and it shows co-morbidity with other diseases, such as anxiety, depression and drug addiction [7]. Although approximately one third of the worldwide population will encounter a traumatic event at least once during their life, only a subset of them develops PTSD (susceptible), whilst most of the trauma victims fully recovers after the first acute physiological response (resilient) [68]. The fact that we cannot predict beforehand who is at risk reflects the fact that the etiopathogenesis of PTSD and the neurobiological mechanisms underlying interindividual variability in susceptibility and resilience still remain largely unknown.

There are also sex differences involved in PTSD susceptibility. The lifetime prevalence of PTSD is two to three times higher in women compared to men. Indeed, after experiencing a traumatic event about 10–12% of women and 5–6% of men develop the pathology [7, 69, 70]. The sex difference in prevalence can be related to several factors. Firstly, men and women are subjected to different types of traumas. Men are more predisposed to assaultive violence (e.g., war, being beaten, shot or stabbed) and accidents than women, while women are generally exposed to more interpersonal and high-impact traumatic events (e.g., sexual assault) [71, 72], and at earlier life stages, compared to men. Moreover, women show different peri-traumatic dissociation (e.g., disconnection, altered awareness, emotional numbing) which may lead to prolonged stress responsiveness [72], and more risk for chronic PTSD development [73]. As previously described by Olff (2017) [69], men and women also have distinct ways of managing stressful situations: (i) women tend to assume more a ‘tend-and-befriend’ response whilst men more a ‘flight-or-flight’ response; (ii) women exhibit more emotional and defensive coping strategies vs. men showing more problem-focused ones; and (iii) women seek more social support from family and friends and they attend more easily psychotherapy than men. Beyond these differences, previous clinical studies evaluated the effects of the menstrual cycle and the underlying hormones on fear memory processes (e.g., fear acquisition, extinction and extinction recall) and on PTSD symptoms and development [74–78]. Particularly, healthy women with lower estradiol levels showed deficits in fear inhibition [79], impaired extinction consolidation [80] and reduced extinction [79] when subjected to fear conditioning procedure, suggesting that estrogen is strongly involved in the regulation of fear. Another study investigated the impact of hormonal status on brain activation during exposure to trauma-related stimuli (e.g., a traumatic film) in healthy women. The findings revealed that neural activation patterns varied according to hormonal levels: women using oral contraceptives showed greater activity in specific brain regions, such as the insula and the dorsal anterior cingulate cortex, compared to women without hormonal contraception [81]. The role of estrogen has been also investigated in women with PTSD but the results are controversial [74]. For instance, Glover and colleagues showed that women affected by PTSD with lower estrogen levels have fear extinction deficits [82], and another study demonstrated that higher estradiol and progesterone levels were linked with increased flashbacks or re-experiencing symptoms in trauma-exposed women [83]. Therefore, although interindividual and sex differences exist in PTSD development, the exact neurobiological mechanisms underlying these differences remain unclear, and preclinical models of PTSD could represent useful tools to address these questions.

## Animal models of PTSD

Animal models are valuable tools to unravel determinants and causative mechanisms underlying stress-related disorders and are crucial for identifying novel pharmacological approaches to treat and prevent the development of such diseases, including PTSD [84–86]. Since PTSD is a very complex psychiatric disorder, numerous paradigms differing in stress type, intensity, duration, and frequency have been proposed to reproduce in rodents the core features of the human pathology in rodents [87–95]. As we previously described [96], PTSD experimental models may consist in the exposure to physical stressors, including footshock [97–102], stress-enhanced fear learning [103–106], single prolonged stress [107–113], underwater trauma [114–117], restraint/immobilization [118–123], as well as social and psychological stressors, such as social defeat stress [124, 125] and predator/predator odor exposure [114, 126–129]. Even though these animal paradigms are able to reproduce in rodents some of the core behavioral symptoms observed in PTSD patients, the majority of them present some limitations that should be improved to obtain higher translatability. For instance, the single prolonged stress/trauma paradigm is able to reproduce the chronic nature of PTSD as symptoms generally occur 7–14 days after trauma [107, 109, 130] and some of them even persist 30 days post-trauma [111]. However, it inadequately captures the multifaceted alterations of memory function, an aspect that is more effectively addressed using footshock-based models. These models, on the other hand, primarily serve to assess cognitive alterations [131] and they are not specific for PTSD [132]; to model PTSD and its chronicity as well, it would need to be paired with risk factors (e.g., social isolation) [98]. Beyond this, a fundamental limitation across most of these PTSD models is their tendency to consider the entire traumatized population as PTSD-affected, without taking into account that not all, but only a small subset of humans, develop the pathology after trauma exposure. These aspects are discussed in the following sections.

## Preclinical models of PTSD: limitations

**Fig. 1:**
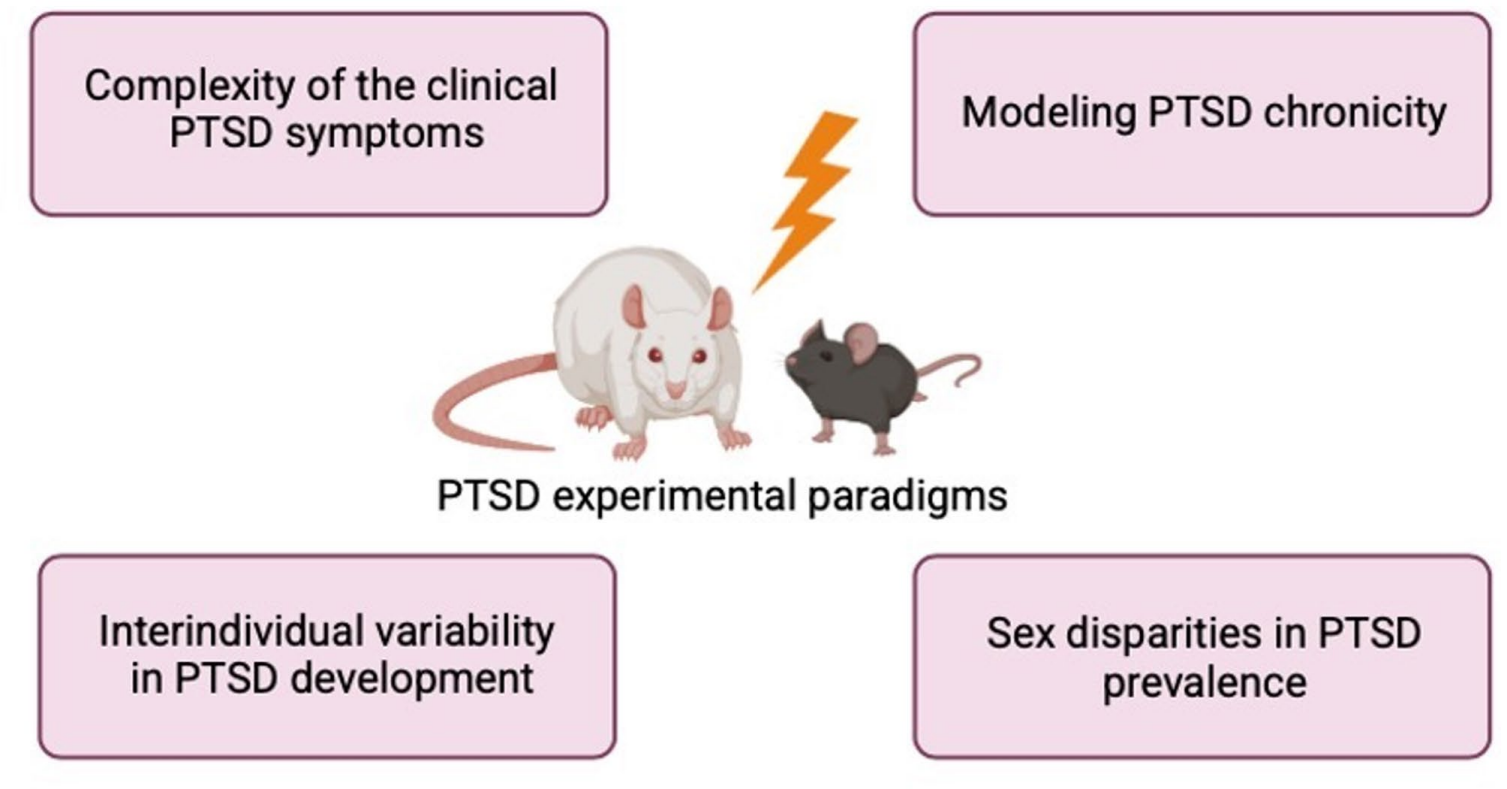
**Limitations of PTSD experimental paradigms**. This figure provides an overview of the main challenges associated with current PTSD models, highlighting aspects where these models may fall short in replicating the complex nature of the disorder

To be valid, preclinical models should meet three criteria: ‘face validity’, ‘construct validity’ and ‘predictive validity’. ‘Face validity’ refers to the ability of an animal model to reproduce the same behavioral alterations observed in patients with the disease. ‘Construct validity’ consists in demonstrating in animal models the same neurobiological underpinnings seen in patients. Finally, ‘predictive validity’ indicates that preclinical models are able to provide a similar response to pharmacological treatments as observed in patients [87, 133, 134]. However, reproducing these three criteria can be very challenging when modeling stress-related disorders such as PTSD [91]. The limitations of PTSD experimental paradigms are represented in Fig. 1, and described in the paragraphs below.

## Complexity of the clinical PTSD symptoms

First, with regard to face validity, there are limitations in reproducing the complexity of the PTSD symptomatology in rodents. According to the DSM-5, PTSD diagnostic criteria are: (A) direct or indirect exposure to an intense source of stress; (B) intrusive symptoms (e.g., trauma re-experiencing); (C) avoidance behavior; (D) negative alterations in cognitions and mood; (E) alterations in arousal and reactivity; (F) duration of symptoms of more than one month; (G) functional significance; and (H) exclusion (symptoms are not caused by medication, substance use, or other illness) [67]. Even though Pavlovian fear conditioning represents one of the most commonly used models for PTSD, it is limited to the evaluation of the cognitive deficits of the pathology only. Briefly, it consists in the exposure to an aversive stimulus (unconditioned stimulus, US; e.g., footshock) paired with a neutral one (conditioned stimulus, CS; e.g., tone or context), and fear memory processes are then evaluated by re-exposing rodents to the CS in the following days [131]. Thus, this behavioral paradigm cannot reproduce the emotional features of PTSD, and it is not highly specific for modeling PTSD as cognitive alterations are common to other psychiatric disorders [132]. However, a way to make the fear conditioning paradigm more suitable to study PTSD is to pair it with risk factors for the disease development. Berardi and colleagues previously developed an experimental paradigm of PTSD, consisting in traumatic experience (footshock) paired with social isolation which it is able to simultaneously captures in rats some of the cognitive (i.e., over-consolidation and reduced extinction of the trauma) and emotional (i.e., reduced sociability) alterations observed in PTSD patients [98, 101].

However, in addition to the emotional and cognitive symptoms of PTSD, there are others that are very difficult, if not impossible, to reproduce. In this context for instance, the manifestation of the intrusive/unwanted symptoms which is described in the PTSD criterion B of the DSM-5 cannot be measured in rodents [91].

## Modeling PTSD chronicity

One of the PTSD diagnostic criteria of the DSM-5 (criterion F) is that the pathology is characterized by a duration of symptoms of more than one month. This aspect is however only rarely considered in PTSD experimental paradigms [91]. By using the Pavlovian fear conditioning, it is not possible to evaluate the PTSD chronicity for several reasons. As described in the above section, fear memory alterations are commonly evaluated in the days immediately following the exposure to footshock (conditioning session), thus over a relatively short period of time [131]. This behavioral procedure then only allows to measure the acute response to stress and fear, making difficult to mimic the chronicity of the pathology.

To effectively study the chronicity of PTSD, a model would need to incorporate extended observation time periods and assessments of long-term behavioral and physiological changes. In a previous study [98], separate cohorts of rats were placed in a context and then subjected to two different stress models: worn cat collar or inescapable footshocks paired or not with social isolation. Interestingly, the exposure to the cat collar induced only an acute fear response, while exposure to footshock caused long-lasting fear memory alterations (even 56 days after trauma), suggesting the importance of the type of trauma in reproducing the chronicity aspect of the pathology. To note the exposure to footshock also affected the emotional domain (e.g., reduced sociability measured in the social interaction task) and such effects were observed only when socially isolated.

PTSD-like symptoms were also evaluated in the long-term in the single prolonged stress paradigm, consisting in rodents being subjected to three different stressors (i.e., restraint stress, forced swim stress, and gas anesthesia until loss of consciousness) in one single session [108]. Most of the studies with this model evaluated PTSD-like phenotype 7–14 days after trauma exposure [107, 109, 110, 130], as the manifestation of the symptoms requires such time interval to develop [109, 113, 135]. Notably, we also recently provided the first evidence that this stress paradigm reproduces some of the PTSD-like alterations long-after (one month) trauma exposure, and that these alterations are sex-dependent [111], indicating that this model is also able to mimic the chronicity aspect as well as trajectories of the pathology.

## Interindividual variability in PTSD development

One of the characteristics of PTSD is the large interindividual variability in stress responsiveness which explains why individuals are either susceptible or resilient after a traumatic event. This represents a critical issue that deserves more attention in PTSD experimental models. Generally, the whole population of the trauma-exposed rodents is considered homogenously, regardless of their phenotypic susceptibility or resilience [30, 114]. However, in order to obtain higher translational value, PTSD animal paradigms should mimic this interindividual variability in the development of the pathology as observed in humans. For this reason, some studies found validated criteria, such as behavioral or other parameters, allowing to separate animals in susceptible or resilient towards the development of PTSD-like symptomatology within the trauma-exposed population (for more details please see [96]). In one study, rats were exposed to two different PTSD models (i.e., predator exposure and underwater trauma) and their increased fear and hyperarousal measured in the elevated plus maze and acoustic startle response tasks, respectively, were used to discriminate rats in susceptible (maladaptive) and resilient (well-adapted) [114]. Further, Ritov and colleagues paired an anxious-like behavior parameter with one associated with depression-like phenotype (i.e., continuous saccharine test) to divide rats, previously exposed to the underwater trauma paradigm, in affected and unaffected [117].

While introducing outcome variability in PTSD models is an improvement, in order to effectively manipulate phenotypes, it is crucial to develop an approach that allows us to predict which animals will develop or not a PTSD-like phenotype after trauma. Identifying these subjects in advance is crucial because it enables the implementation of targeted preventive or therapeutic interventions. This approach involves finding reliable indicators or biomarkers spanning from before to after trauma that can predict an individual’s vulnerability to developing the pathology. Therefore, finding a predictive variable has a dual advantage as it can give better knowledge on the underlying mechanisms of PTSD susceptibility and it can help in the development of more effective and personalized pharmacological treatments. In this scenario, Olson and colleagues used the acoustic startle response measured before and after trauma to discriminate between susceptible and resilient mice, and they found that only the susceptible ones then exhibit aggressive behavior and reduced sociability in the resident-intruder and social interaction tasks after trauma, respectively [136]. A similar method based on the arousal measurement was used in another study, which showed that only susceptible (not resilient) mice previously exposed to restraint stress later developed some of the emotional and cognitive PTSD-like alterations long after trauma [123]. Instead of using predictive variable related to the emotional domain, two previous studies found that rodents with increased freezing behavior during the intertrial interval of a stress-enhanced fear learning paradigm showed emotional and cognitive PTSD-like symptoms [103, 137]. Even more differently, exploratory activity of a novel environment five days after trauma (i.e., footshock paired with social isolation) has been identified as a predictive variable for susceptibility (low exploratory activity) or resilience (high exploratory activity) to the later development of PTSD-like alterations (i.e., over-consolidation, increased retrieval and reduced extinction of the traumatic memory, and socio-emotional deficits) in both male [99] and female [138] rats. Beyond using behavioral parameters as predictive variables, more recently, a blunted glucocorticoid responsiveness measured after prepubertal stressors significantly correlates with PTSD-like alterations (i.e., reduced fear extinction, small hippocampal volume and rapid-eye-movement sleep’s disturbances) [139].

Therefore, considering susceptible and resilient phenotypes separately is critical for the evaluation of the neurobiological and causative mechanisms of PTSD which nowadays are still unclear. The lack of consideration for interindividual variability hampers the ‘construct validity’ as well as the ‘predictive validity’ criteria of PTSD experimental models, as the incomplete understanding of the neural circuits and molecular pathways involved in the disease does not allow to have a gold standard pharmacological therapy yet. Current treatments approved by U.S. Food and Drug Administration (FDA) and often prescribed to PTSD affected patients include serotonin selective reuptake inhibitors (SSRIs), such as sertraline and paroxetine. However, these drugs are only partially effective and they reduce the anxiety symptoms of PTSD without treating the cognitive ones [140, 141].

Given that the current pharmacological treatments used for PTSD do not act on the cognitive symptoms, additional methods have been provided for this purpose. For instance, psychological interventions (e.g., prolonged exposure therapy) consist in helping patients confront trauma-related memories, emotions, and situations they have been avoiding. By repeatedly re-exposing them to these trauma cues in a controlled and therapeutic environment, the therapy aims to reduce the emotional distress associated with the traumatic memories and lessen PTSD symptoms over time [142]. As these methods are characterized by high drop-out rates and relapse [143, 144], to increase their efficacy a combination of psychotherapy and pharmacotherapy has been evaluated to treat PTSD [145]. Given the role of the endocannabinoid system components as biomarkers of PTSD development [32], it has been previously demonstrated that treatment with the synthetic cannabinoid URB597 after the extinction sessions at 7, 10 and 13 post-trauma days (modeling the exposure therapy) restores long-term cognitive and emotional PTSD-like alterations [101]. Similar results were found in another study but here rats were chronically treated with URB597 starting from one week after trauma and the extinction sessions performed for four consecutive days [146]. In addition to research on endocannabinoid-based drugs, numerous preclinical and clinical studies have explored the effects of glucocorticoids on fear memory processes. For example, in animal models, glucocorticoid administration has been shown to enhance both the extinction learning and reconsolidation of fear memories [147], which may be due to the increased GR signaling in the brain of traumatized rats treated with corticosterone [148]. Elevated cortisol levels appear to support these processes by activating physiological stress responses essential for effective PTSD treatment engagement [149]. Consistently, clinical studies in PTSD patients indicate that glucocorticoid administration combined with prolonged exposure therapy not only promotes fear extinction but also improves treatment retention [150, 151].

However, most of the PTSD experimental models are designed to study the effects of pharmacological rather than psychological interventions, which represents another limitation of these animal paradigms. Therefore, this indicates that there is an urgent need to improve the experimental models in order to better mimic the human condition.

## Sex disparities in PTSD prevalence

The aspect of the biological sex in preclinical studies on PTSD, and more widely in the neuroscience field, represents another important limitation. The paucity of studies carried out in female subjects could be due to several reasons. First of all, some behavioral paradigms have been specifically designed for males. For example, reproducing bullyism (and thus fighting) in female rodents has been challenging for years due to the strong differences in social behavior and aggression patterns between male and female animals. Only recently social defeat stress models suitable for female mice have been developed and they are able to induce several behavioral alterations [152, 153]. Using female rodents in behavioral experiments is highly complex also because of their estrous cycle and underlying hormone fluctuations. The fluctuations in sex hormones over the 4 days estrous cycle significantly influence various processes, including the activity of the HPA axis. This hormonal variation therefore introduces additional variables that must be controlled for or accounted for the experimental design. This would require a significantly higher number of animals to perform experiments according to each phase of the estrous cycle, which conflicts with the principles of the 3Rs (replacement, reduction and refinement) aiming at minimizing animal use in research. Moreover, it could be very challenging in cases where, for example, the experimental timeline is lengthy. Considering the estrous cycle phases is therefore important when studies are specifically aimed at assessing the outcomes of a particular factor (such as stress) in relation to different female hormones levels. However, whether the vulnerability for developing stress-related disorders is related to the specific hormonal cycle phase at the time of trauma is less investigated. In this context, Velasco and colleagues found no differences on fear memory alterations between female mice subjected to trauma under high (proestrus) and low (estrous) hormones levels [154]. These results may suggest that the different hormones levels at the moment of trauma do not influence the vulnerability for stress-related alterations, and that therefore examining the response of the entire female population without considering estrous cycle could be also of higher translational value when mimicking stress-related disorders in rodents. This would help in overcoming all the previously described limitations in the use of female rodents in the stress field as well as it would enhance the translational value of the preclinical studies, as it would more reflect what occurs in humans (since hormones levels are not monitored when a woman experiences a trauma). Beyond this aspect, while in human studies it has been indeed established that women are more likely to develop PTSD [7, 69, 70], this gender bias is not replicated in rodent models. This inconsistency may stem from the inability of rodent models to adequately capture the full complexity of trauma experience in humans. As a result, it becomes challenging to investigate and understand the biological mechanisms that contribute to the differing prevalence rates of PTSD between women and men. Addressing these limitations is crucial for advancing our knowledge and developing more effective treatments.

## Conclusions

Sex differences are a main factor that underlying the interindividual variability in PTSD susceptibility. Although women are more likely to develop stress-related disorders, such as PTSD, female rodents have been overlooked in preclinical studies, so far. It is urgent to start designing PTSD paradigms in both male and female rodents to further decipher the sex-dependent biological mechanisms in stress-related disorders.

In particular, researchers must implement rigorous standards for the inclusion of sex and gender variables in study design, analysis, and reporting. This involves systematically treating sex as a biological variable (SABV) and applying sex- and gender- based analysis (SGBA) [155, 156]. For example, experiments should be well-designed and should include both male and female subjects with adequate sample sizes to allow for statistically meaningful comparisons. Without rigorous experimental planning and robust statistical analyses, studies may lack sufficient statistical power, and critical insights into the biological impact of sex could be missed. Moreover, the use of appropriate statistical tests is strongly recommended, such as two-way ANOVA with sex and intervention (e.g., stress exposure) as independent variables. This approach allows researchers to detect not only the main effects of sex and stress but also their interaction, thereby enabling the investigation of sex-dependent effects on stress responses [156].

Alongside these considerations, distinguishing between susceptible and resilient individuals is essential to better understand the underlying mechanisms triggering the development of PTSD in the subset of susceptible individuals. This could lead to novel biological insights and the identification of biomarkers for PTSD vulnerability, ultimately allowing the development of personalized and effective therapies to treat and cure PTSD, whose prevalence is highly increasing. Such approaches will benefit not only females, who are at higher risk of developing PTSD, but also males, thereby advancing the goals of precision medicine for all individuals.

## Data Availability

No datasets were generated or analysed during the current study.

## Abbreviations

ACTH: Adrenocorticotropic hormone
AR: Androgen receptor
CBG: Corticosteroid binding globulin
CRH: Corticotropin-releasing hormone
CRHR1: CRH receptors type 1
CRHR2: CRH receptors type 2
CS: Conditioned stimulus
DSM-5: Diagnostic and Statistical Manual of Mental Disorders
ERα: Estrogen receptor alpha
ERβ: Estrogen receptor beta
GR: Glucocorticoid receptor
HPA: Axis hypothalamic pituitary adrenal axis
HPG: Axis hypothalamic pituitary gonadal axis
MR: Mineralocorticoid receptor
PR: Progesterone receptor
PTSD: Post-traumatic stress disorder
PVN: Hypothalamic paraventricular nucleus
SABV: Sex as a biological variable
SGBA: Sex and gender-based analysis
US: Unconditioned stimulus
